# Measuring local-directional resolution and local anisotropy in cryo-EM maps

**DOI:** 10.1038/s41467-019-13742-w

**Published:** 2020-01-02

**Authors:** Jose Luis Vilas, Hemant D. Tagare, Javier Vargas, Jose Maria Carazo, Carlos Oscar S. Sorzano

**Affiliations:** 1 Biocomputing Unit, Centro Nacional de Biotecnologia (CNB-CSIC), Campus Universidad Autonoma, 28049 Cantoblanco, Madrid Spain; 2 Department of Biomedical Engineering, Yale University, New Haven, CT 06520 USA; 3 Department of Anatomy and Cell Biology, McGill University, Montreal, H3A 0G4 Canada

**Keywords:** Computational biology and bioinformatics, Cryoelectron microscopy

## Abstract

The introduction of local resolution has enormously helped the understanding of cryo-EM maps. Still, for any given pixel it is a global, aggregated value, that makes impossible the individual analysis of the contribution of the different projection directions. We introduce MonoDir, a fully automatic, parameter-free method that, starting only from the final cryo-EM map, decomposes local resolution into the different projection directions, providing a detailed level of analysis of the final map. Many applications of directional local resolution are possible, and we concentrate here on map quality and validation.

## Abstract (web summary)

It is important to analyse the local resolution of cryo-EM maps. Here the authors present MonoDir, a fully automatic and parameter free method for the directional local resolution analysis of cryo-EM maps that requires only the final map as input and they also propose indicators for assessing map quality.

## Introduction

Once a map is reconstructed, its resolution is estimated to avoid possible over-interpretations. Resolution can be determined in a global or in a local manner. The global one is a single parameter that determines the degree of reliability of the whole map; being the FSC the most common (for a review of resolution measures see ref. ^1^). In contrast, local approaches determine this reliability voxel by voxel through different approaches as windowed FSC between two halves^2^, or the detection of structural features above the noise level^3,4^.

The need for local resolution approaches emerges as a consequence of multiple effects happening at the sample at the imaging and processing levels, such as: existence of sample heterogeneity, flexibility, wrong angular assignment of single particles, bad angular coverage of the projection sphere, existence of preferred directions or radiation damage among others. Local resolution maps may quantify the combined effect of all these limiting factors into the final cryoEM map; however, they are not able to isolate the effect of each factor alone. Recent works like the 3DFSC^5^ or the efficiency^6^ have tried to address individually some of these problems introducing the concept of directional resolution. However, these approaches were intrinsically global, determining a merit function that informs on the existence of a bad angular coverage. Local measures of these effects have not been studied yet, being this issue one of the goals of our study and, up to our knowledge, the first time directionality and locality have been brought together. In this work, a local-directional resolution measurement approach is proposed. We will show here that, as opposed to just local resolution methods^2–4^, by expanding the concept of (voxel) locality by performing a complete “directional local resolution” analysis, other way to analyze the informational content of a cryo-EM map emerges, opening many new possibilities, from map validation and quality control to chain tracing, among others. This article focuses on some of the suggested quantitative indicators of map quality assessment. In all cases we want to note two important points: (1) that the single input to our method is the final cryo-EM map, without any knowledge of the original particles or their assigned projection directions, (2) that our approach rests on the strong mathematical framework of monogenic signals^7^, building on MonoRes^4^ (see Methods section).

The new algorithm, that we call MonoDir, calculates the local resolution along a set of directions in 3D (see Methods section). In essence, given a voxel location (${\bf{r}}$), we calculate the contribution to its aggregated local resolution of the information coming from each direction. In other words, each location (voxel) will have associated to it a set of resolution values equal to the number of analyzed directions; they are called local directional resolution values. Note that, essentially, what we are doing is to treat local resolution not just as a single “value”, the directional resolution operator assigns a real number to every direction at every voxel, the changed is, therefore, of a very fundamental mathematical nature (thus it is a function on the product space ${{\mathbb{R}}}^{3}\,\times\,R{P}^{2}$ where the ${{\mathbb{R}}}^{3}$ component is the voxel and the $R{P}^{2}$ component is the direction. Previously defined local resolution operators are functions only on ${{\mathbb{R}}}^{3}$. Extending the notion of local resolution to ${{\mathbb{R}}}^{3}\times R{P}^{2}$ is the main contribution of this paper.). Consequently, it is no wonder that MonoDir offers the possibility to analysis cryo-EM maps from totally new perspectives, much beyond the limited set of examples we present in this work, all oriented towards map validation.

The simplest quality indicators we can think of are “the Highest (e.g., 3 Å) and Lowest (e.g., 10 Å) local-Directional Resolutions Maps”. The highest resolution map informs about the highest resolution value, $H({\bf{r}})$, for each pixel, ${\bf{r}}$, without taking into account the direction along which that resolution is measured. Similarly, the lowest resolution map can be obtained with the corresponding lowest resolution values, $L({\bf{r}})$. In general, the highest and lowest resolution directions do not need to be orthogonal to each other. To get statistically “robust” maps, the values of $L({\bf{r}})$ and $H({\bf{r}})$ are considered as the percentiles 0.05 and 0.95, respectively, of the resolution distribution of all possible directions.

The next indicator that we have introduced is “the Average Directional Resolution” (ADR). It only requires the detection of the direction contributing to the highest resolution $H({\bf{r}})$ (and which is this maximum resolution value) and, in the same way, to the lowest resolution $L({\bf{r}})$, both in the robust statistical sense previously mentioned. The ADR is mathematically defined as:

1 $${\mathrm{ADR}}({\bf{r}})\,=\,\frac{L({\bf{r}})\,+\,H({\bf{r}})}{2}.$$

The ADR provides valuable information about the map quality and informs about the possible existence of preferred directions. However, to infer this information it is required the local resolution map to compare with, the ADR map by itself is not a local anisotropy metric. In a way, it is like a local resolution map weighted by the local anisotropy. Note that when the values $L({\bf{r}})$ and $H({\bf{r}})$ are similar, it means that the map presents a low local resolution anisotropy, and therefore all directions exhibit the same quality. In contrast, if there is local anisotropy, the value of the lowest resolution will be substantially smaller than the one of the highest resolution, making the mean lower. To complement ADR and getting an estimator of resolution anisotropy, a dispersion metric is introduced, thus, for each voxel we introduce a statistically robust metric, the half interquartile range between the 0.17 and 0.83 percentiles (these percentiles are selected to represent an unit of standard deviation when the distribution is normal). It is reminded that for each voxel, MonoDir computes the local-directional resolution for a set of directions; therefore, there exists as many local-directional resolutions as number of computed directions. The percentiles are calculated from this distribution of local-directional resolutions. It must be highlighted that as dispersion metric, the half interquartile range is therefore our proposed metric for measuring the local resolution anisotropy.

**Fig. 1:**
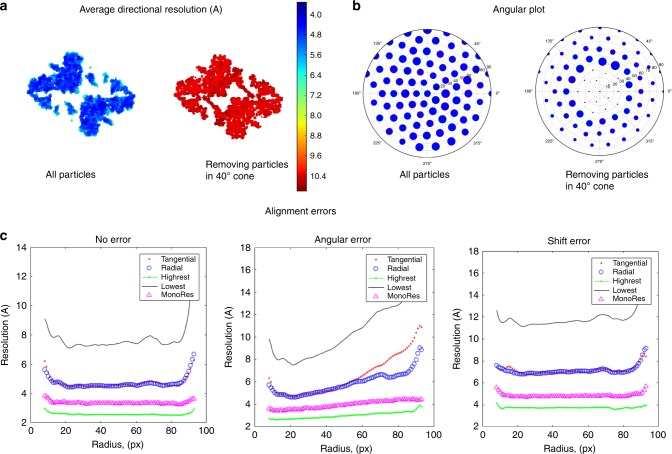
Results from a computer simulated map from the beta-galatosidase. The atomic model PDB-3j7h was converted into a density map, first for maps obtained considering all projection directions and then with missing directions, showing (**a**) Average Directional Resolution (ADR), (**b**) angular plot, (**c**) radial average of local-directional resolution maps (tangential-pink points, radial-blue circles, as well as lowest and highest) when: random angular errors of standard deviation of 1.2 degrees, random shift errors of standard deviation of 1 degrees, and error free reconstructions are considered.

Obviously, the ADR and the half interquartile range inform about the resolution anistropy, but they do not inform about which are the preferred directions. However, this piece of information can be very easily measured by introducing a modification of the otherwise classical plot of projection directions. The new plot will be referred to as polar plot and it represents the number of voxels that have their highest resolution along a specific direction. In other words, the polar plot represents a histogram of the highest local-directional resolution values on the projection sphere, where the number of counts along each direction is represented by the size of the dot, see an example in Fig. 1. Thus, we are capable of identifying not only situations of systematic lack of particle views (as normal polar plots do), but errors in angular assignment as well. Indeed, if our 3D alignment algorithm had incorrectly assigned to a certain projection direction images that were really coming from a different direction, this error will introduce blurring in the map along that direction and, consequently, the number of voxels with the highest resolution will decrease. Therefore, our new angular plot should present an uniform coverage of the projection sphere for isotropic maps, otherwise we either have a lack of projection directions or a bad angular assignment.

MonoDir computes the local-directional resolution map along a certain set of directions. However, it is also possible to analyze the local directional resolution along particular directions that may be related to critical characteristics of the map. In this context, it is specially interesting to analyze resolution along two directions connected to a polar decomposition of the map: the radial and tangential directions. In the case of the calculation of the radial local resolution, for each point of the map we calculate the direction of a line joining that point to the center of the map, and we calculate the local resolution along this direction, see Supplementary Fig. 1. For the case of the tangential resolution, at each point in the map we calculate its tangential plane and we average all directional resolutions in that plane. The expectation is that these directions would be particularly sensitive to errors in angular alignment, specially when resolution along those directions is plotted along radii. Specifically, an approximately linear relation between radius and radial average of resolution can be observed, being the slope related to the angular error in the angular alignment (as expected by simple geometrical considerations, at least for the tangential resolution). This is described in depth in the Supplementary Information. Note that angular plot and the radial average resolution curves show global information about the reconstructed map, but it would never obtained without calculating first the associated local directional information.

## Results

### Local-resolution anisotropy: synthetic map

We first tested MonoDir in a synthetic fully controlled setting, using the structural model of $\beta$-galactosidase corresponding to PDB entry $3j7h$^8,9^. The aim of this synthetic example is to illustrate in a detailed manner the information that MonoDir provides. Thus, the atomic model was first converted into an electron density map^10^ and a gallery of 500 random projections was obtained with known angular assignments; Gaussian noise with zero mean and standard deviation of 2 a.u. was then added. We performed two experiments with these data, the results are presented in Fig. 1. The first experiment aimed at simulating the case of having preferred orientations on the cryo-EM grid, so we removed all particles with tilt angles smaller than 40 degrees (missing cone). The lack of information along the missing directions naturally introduced a resolution anisotropy in the map. Moreover, MonoDir works by establishing statistical tests to elucidate if the local energy of signal is higher than the energy of noise. Along the missing cone direction, there is nothing to compare, and the resolution assigned is the lowest possible. The second experiment obtained the reconstructed structure without removing any particle orientation, this experiment addressed the relationship between the radial profiles of radial and tangential resolution and the presence of alignment errors. In this way, we analyzed these plots for the cases of exact refinement (no errors), in-plane shift errors only, and, finally, angular errors only.

To start with the ADR, the values were clearly different between the map reconstructed from all particles Fig. 1a and the one obtained in the case of a missing angular gap Fig. 1a. As expected, the value of ADR was much lower in the presence of anisotropy than in its absence. Then, we tested the behavior of the new polar plot of projection directions, showing the number of voxels with the highest resolution per direction Fig. 1b. Clearly, the plot in (1b), corresponding to the case with no angular gap (b-on the left), is much more even than the case showing an angular projection gap (b-right). In the latter case the angular gap, forming a cone of 40 degrees, is very clear in the center of the plot (the projection directions around the missing cone have a somehow exaggerated number of voxels with the highest resolution, an effect we have noticed in transition regions and obviously is a consequence of the synthetically-imposed missing cone, therefore, this effect should not occur in experimental cases).

Finally, the plots of radial and tangential resolutions per radius are very informative of alignment errors and confirm our previously introduced expectations; to test this issue, the known angular assignment of particles and shift were randomized around the true direction; a Gaussian distribution with a standard deviation of 1.2 degrees was considered for the angular assignment and a standard deviation of 1 pixel for the shift. In this way, the plot corresponding to the case of not having errors in the assignment of projection directions Fig. 1 (c, left) shows flat profiles and a highest directional resolution close to Nyquist. Then, the plot where only shift errors were introduced Fig. 1 (c, center) also have flat profiles, but with values shifted to lower resolutions. Finally, when angular errors were introduced, a clear slope appears Fig. 1 (c, right), indicating the existence of angular errors (and their importance—larger errors induce a larger slope). Note the similar behavior of the tangential and radial resolutions at lower radii, roughly corresponding to the maximum sphere fully contained inside the macromolecule, while they diverge beyond this point, with the tangential resolution normally having a higher slope. It is also very clear how both the lowest and highest directional resolution estimations also follow a clear slope with the radius, being MonoRes estimation always close to the maximum resolution, although lower (a logical behavior considering how it is defined).

### Local-resolution anisotropy: experimental maps

**Fig. 2:**
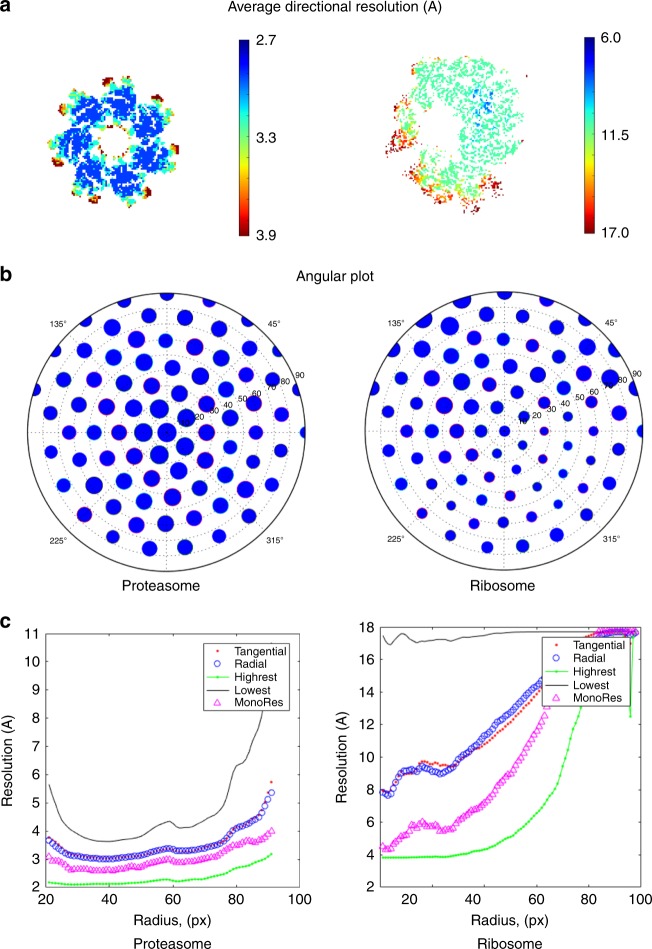
Results for proteasome 20S and ribosome 80S. **a** Average Directional Resolution (ADR), (**b**) polar plot, and (**c**) radial average of local tangential (pink points) and radial (blue circles) directions plotted along radii, together with the highest (green points), lowest (black continuous) and MonoRes (violet triangles) local resolution estimations.

We start with the case of two data sets, the 20S Proteasome^11^ (EMDataBank: EMDB-6287) and the 80S Ribosome^12^ (EMDB-2275), which are shown side by side in Fig. 2. Starting with the ADR (Fig. 2a), the values for the proteasome all over the map are much lower than those for the ribosome, indicating that the proteasome map is much more isotropic. The comparison between ADR values and the FSC also support this observation, since they are similar to the reported FSC resolution of $2.8$  Å (at 0.143); in addition, another indicator, the 3DFSC^5^ (see Supplementary Figures) also indicates isotropy. On the contrary, the case of the ribosome is totally different, with a wide dispersion of ADR values. Then, polar plots for directional distribution of particles (Fig. 2b) show a quite even distribution for the proteasome, but a very uneven distribution of highest resolution directions for the ribosome. Finally, note that plots of radial and tangential resolutions along radii are very informative (Fig. 2c), with those coming from the proteasome presenting a quite small slope—an indicator of a good angular assignment—while those calculated for the ribosome show a very high slope. We note that this ribosome sample was also used as example in ref. ^6^, reaching also the conclusion that the angular alignment was imperfect, but the new insight is that we have been able to prove it without any knowledge of the original images or their angular assignment (as the method in ref. ^6^ needed).

**Fig. 3:**
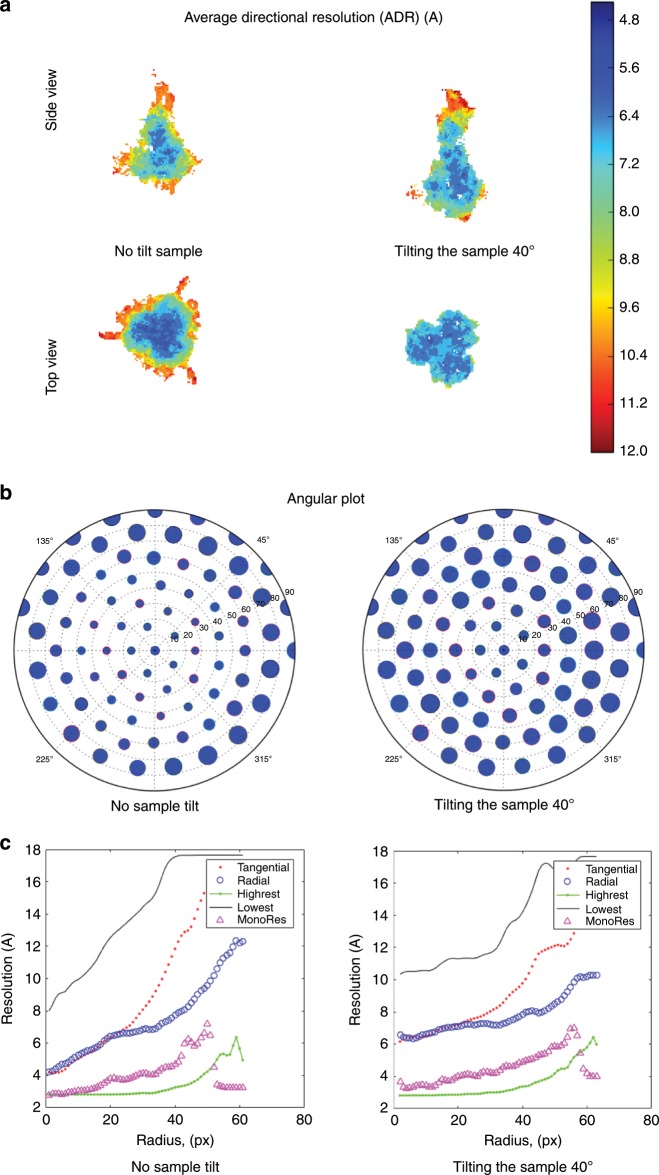
Results for Influenza Hemagglutinin (HA) trimer when the sample is untilted and tilted. **a** ADR, (**b**) angular plot, and (**c**) radial averages as in Fig. 1.

The last experimental example of MonoDir application is presented in Fig. 3. In this case, the specimen on the left and right columns is the same, Influenza Hemagglutinin (HA) trimer^5^, for which two maps were deposited (unfiltered maps at 0 and 40 degrees in EMPIAR entries 10196, 10197, respectively), the first one was strongly affected by the presence of preferred orientations, while this handicap was greatly minimized in the second map by tilting the EM grid. It is clear in Fig. 3a, left uncorrected map, right corrected, that ADR values are higher for the uncorrected map than for the corrected one. The success of the map correction was initially supported by the 3DFSC^5^ (see Supplementary Figures 3DFSC and dispersion). The polar angular distribution plots (Fig. 3b) exhibit a non-uniform distribution of highest local-directional resolutions, indicating the existence of preferred directions, top views in this case. In contrast, for the tilted sampled, the same plot presents a more uniform local-directional resolution coverage of the projection sphere, although with a slight but not negligible non-uniform coverage inside a central cone. Finally, radial plots of radial and tangential resolutions (Fig. 3c) indicate a higher slope for the uncorrected map than for the corrected map, exactly as expected.

## Discussion

We have further expanded the concept of “local resolution” to “directional local resolution”, where the contribution to the local resolution estimation associated to different directions is individually analyzed. The concept of resolution depends not only on the map position, but having different values at different directions. This fact adds a new dimension to the capacity to study a cryo-EM map. In this article we have introduced some “map quality indicators”, but many more could be imagined, and in completely different application domains, such as, automatic chain tracing or quality checks within iterative angular alignment algorithms. We present, therefore, a very general tool and its initial application to map validation, but we expect this tool to have a much wider impact in cryo-EM.

We have also shown how the use of local-directional measurements provide new information about the quality of the reconstructed map, identifying angular alignment errors and the existence of preferred directions and resolution anisotropy; moreover, this information is extracted from the reconstructed map, without the need to access the original set of images or any other information related to them (this is a unique and very strong feature of *MonoDir* that no other methods posses). Regarding the capacity of *MonoDir* to identify alignment errors, we have presented clear examples in which the slope of radial plots of both tangential and radial can be qualitatively related to the existence of higher of lower angular errors, providing a unique guidance tool that did not exist in the field before. Furthermore, in Supplementary Information we have analyzed more in-depth this effect, proving that the approximation to a linear behavior between alignment error and tangential and radial resolution slopes indeed hold, although more work is need to fully characterize this effect, probably analyzing scaling terms not yet taken into account, so that ultimately we could provide with a quantitative description of this relationship.

In conclusion, the set of examples analyzed in this work with *MonoDir* represent a proof of concept of how important is to treat local resolution not as a scalar but as a directional measurement. In all cases *MonoDir* was able to provide a new view of the cryoEM map, helping us to understand better its quality by providing an unprecedented level of detail regarding the source of the possible errors affecting the map. In this respect, we consider that the possibilities of “directional local resolution” are only starting, and that future works will propose novel extensions applicable to many areas of work in the field.

## Methods

### MonoDir algorithm

The root of *MonoDir* is *MonoRes*^4^ in which an estimation of the monogenic signal and its local amplitude at different frequencies was used to determine by an hypothesis test on the cryo-EM map whether signal (macromolecular structure) estimation is significantly higher than noise estimation at any given frequency. A mask allows to discriminate between noise and protein. To measure local-directional resolution maps and, therefore, anisotropy, a directional filter must be previously applied.

Our goal was to filter directionally the electron density map and then to calculate the local resolution of the directional-filtered map obtaining the so-called local-directional resolution map. In Supplementary Figures a scheme of the measurement process is shown. The directional filter is defined by determining the Fourier Transform of the volume and masking the Fourier Transform with a cone that only keeps the frequencies inside it. The cone axis is arranged along the direction in which the local-directional resolution is calculated, and the cone angle is set by the user (in our examples we used 30 degrees). By computing the inverse Fourier Transform, a directional filtered map is obtained.

We then measured the local resolution of these directionally-filtered maps using *MonoRes*^4^. MonoRes computes the local amplitudes of noise and signal. Unfortunately, despite using smooth filters in Fourier space, the directional filtering introduced some ringing, in particular around the borders and in the plane perpendicular to the measured direction. This ringing might modify the noise statistics. To avoid that, the original MonoRes algorithm was modified. These modifications can be found in the Supplementary Information.

The process of measuring local-directional resolution is repeated until we have covered the full projection sphere. In our examples, we computed 81 directions with an angular sampling of 15 degrees. Once all directions were analyzed, the local-directional resolution information is combined to produce the different outputs (lowest, highest, radial/tangential resolution maps, and the local average directional resolution map).

## Supplementary information

Supplementary Information

## Data Availability

All published data sets used in this paper were taken from the Electron Microscopy Data Bank (https://www.ebi.ac.uk/pdbe/emdb/) and, their entries are: EMD-2984, EMD-2275, EMD-6287, and EMD-8731 (last one also in EMPIAR (https://www.ebi.ac.uk/pdbe/emdb/empiar/) entries 10097 and 10096).

## Code availability

MonoDir is publicly available in Xmipp^13^ and has been integrated in Scipion^14^ (http://scipion.cnb.csic.es).

## References

[1] Sorzano COS (2017). A review of resolution measures and related aspects in 3D Electron Microscopy. Progress Biophys. Mol. Biol..

[2] Cardone G, Heymann JB, Steven AC (2013). One number does not fit all: mapping local variations in resolution in cryo-EM reconstructions. J. Struct. Biol..

[3] Kuculkelbir A, Sigworth FJ, Tagare HD (2014). Quantifying the local resolution of cryo-EM density maps. Nat. Methods.

[4] Vilas JL (2018). MonoRes: automatic and accurate estimation of local resolution for electron microscopy maps. Structure.

[5] Tan YZ (2017). Addressing preferred specimen orientation in single-particle cryo-EM through tilting. Nat. Methods.

[6] Naydenova K, Russo C (2017). Measuring the effects of particle orientation to improve the efficiency of electron cryomicroscopy. Nat. Commun..

[7] Unser M, Sage D, Van De Ville D (2009). Multiresolution monogenic signal analysis using the Riesz-Laplace wavelet transform. IEEE Trans. Image Process..

[8] Bartesaghi A (2014). Structure of $$\beta $$ -galactosidase at 3.2-A resolution obtained by cryo-electron microscopy. PNAS.

[9] Bartesaghi A (2015). 2.2 Å resolution Cryo-Em structure of beta-galactosidase in complex with a cell-permeant inhibitor. Science.

[10] Sorzano COS (2015). Fast and accurate conversion of atomic models into electron density maps. AIMS Biophys..

[11] Campbell MG, Veesler D, Cheng A, Potter CS, Carragher B (2015). 2.8 Å resolution reconstruction of the Thermoplasma acidophilum 20S proteasome using cryo-electron microscopy. Elife.

[12] Bai XC, Fernandez IS, McMullan G, Scheres SHW (2013). Ribosome structures to near-atomic resolution from thirty thousand cryo-EM particles. Elife.

[13] de la Rosa-Trevín JM (2013). Xmipp 3.0: an improved software suite for image processing in electron microscopy. J. Struct. Biol..

[14] de la Rosa-Trevin JM (2016). Scipion: a software framework toward integration, reproducibility, and validation in 3D electron microscopy. J. Struct. Biol..

