# Supplementary material for: Measuring local-directional resolution and local anisotropy in cryo-EM maps
Source: Nat Commun. 2020 Jan 2;11:55. doi: 10.1038/s41467-019-13742-w (PMC6940361; doi:10.1038/s41467-019-13742-w)
Supplement: Supplementary file 1 — Supplementary Information [file 41467_2019_13742_MOESM1_ESM.pdf]

Supplementary Information: Measuring local-directional resolution and local anisotropy in cryo-EM maps

J.L. Vilas et al.

Supplementary Information

Supplementary Note 1: Modifications on MonoRes core for MonoDir algorithm

The non-directional *MonoRes* algorithm computes the non-directional local resolution of a given map by means of statistical tests at different frequencies. The method begins by high pass filtering the map with center frequencies ranging from low to high, and calculating the local monogenic amplitudes of the map at the center frequency. *MonoRes* statistical tests attempt to determine if the local amplitudes at the filtering frequency are significant higher than the 95th percentile of the distribution of noise. If they are, then the voxel is declared to have significant amplitude at the filtering frequency. The highest filtering frequency at which a voxel has significant amplitude is declared to the local resolution of the voxel. The distribution of noise is calculated by considering all voxels outside a particle mask. To avoid the existence of false positive, a local resolution value is assigned to a voxel when the hypothesis test fails consecutively twice, being the assigned resolution value is the last frequency that passed the hypothesis test.

To introduce directional measures, the input map is directionally filtered along of a set of directions that cover the projection sphere. The directional filter is carried out in Fourier space by means of cones. Thus, for each directionally filtered map the estimation of local resolution maps can be performed by applying *MonoRes*. Unfortunately, the use of directional filters introduces artifacts that might affect to the local resolution that *MonoRes* will estimate. This kind of artifact is due to the filter-ringing that the directional filter generates. There are two critical modifications:

1) The particle radius of the protein is initially determined (radius of the sphere that contains the whole protein). Then, to obtain the noise statistics, we only look outside of this sphere, see Suppl Fig. 2.

2) All points inside the shell are not valid, note that directional filters introduce a ringing in the orthogonal direction to the filtering direction. Therefore, the distribution of noise is estimated in the intersection of the shell with the cone defined by the filtering direction. See Suppl Fig. 2.

Other modifications of the *MonoRes* algorithm were carried out in terms of performance. Note that to compute the resolution anisotropy, it is necessary to apply *MonoRes* as many times as the number of analyzed directions. As a consequence, *MonoRes* algorithm was carefully re-implemented and adding a thread parallelization in an efficient manner.

Supplementary Note 2: Angular assignment errors

In the main text it was shown how the radial averages of radial and tangential resolution can be used to identify angular assignment errors in the reconstructed maps. However, it remains to relate the slope of the radial average and the committed error. To do that, two atomic models were considered, the first one was the $\beta-$galactosidase [4] with pdb entry $5a1a$ and the second one was the ribosome [5] with pdb entry $5wf0$. Both models were converted into density maps using [6] and a set of 500 projections were generated with a sampling rate of 1 Å/pixel. Then, noise was added to the set of projections with zero mean and standard deviation of 2 a.u. Because of the way projections were generated, the angular orientation of the set of particles is well known. However, to introduce angular assignment errors, the angles were randomized following a normal distribution with a given standard deviation. The choice of this standard deviation establishes the angular assignment error, note that the 99% of the distribution will be in the interval $[-3\sigma, 3\sigma]$ (if $\sigma = 1$ degree, the maximum committed error will be 3 degrees). Thus, synthetic maps reconstructed with angular assignment errors corresponding with $\sigma = 1, 1.5, 2, 2.5, 3$ degrees were considered and evaluated with *MonoDir*.

The analysis of the angular assignment error is carried out with the radial average curves. In Fig. 6 the *MonoDir* results for the radial and tangential components are shown. Note that the higher σ (angular assignment error), the greater slope of the radial average curves.

In Suppl. Fig. 6(a) the results with $\beta-$galactosidase can be observed. Figure shows that the behaviour of the radial averages of the radial and tangential components is different. In particular, both curves diverges at specific radius, in particular at 20px, 40px and 60 px. This is easily explained considering the shape of the protein. The reason for these divergences is the macromolecule geometry, see Suppl. Fig. 7. The $\beta-$galactosidase has a hole with radius of 20px in the center and, therefore, the region 0-20 px does not have structural meaning. From 20 px to 40 px, there exist voxels with structure along all possible directions and, in this region, the radial averages present a linear behaviour. However, when the radius is greater than 40 px, and because the structure is very elongated, there is no structure for averaging along one of the axis, and therefore, the radial resolution cannot be measured properly. The results is that the radial average curve is affected, changing its behaviour. The same scenario occurs at 60 px, which represents the second divergence point of the radial averages. The conclusion we derive from these experiments is very simple: protein geometry affects the radial averages, so that the slopes of these curve must be measured in those regions in which there is mass essentially in all directions, which ensure the proper measurement of the radial component responsible of this effect. Summarizing, the deviations from the linear behaviour of the radial average curve for the radial and tangential resolutions are due to the protein geometry. To check that, the structure of the ribosome was chosen because it is relatively homogeneus in all directions. Furthermore, to reduce possible problems with the measurement of the radial resolution component, the 500 particles were circularly masked (with radius 80 px), ensuring that inside the circle there always exists informational content, then noise was added and the map reconstructed. In Suppl. Fig. 6(b) the radial averages of the masked ribosome as result of *MonoDir* are shown. Note that the geometry of the masked ribosome is spherical and therefore, the radial average curve does not present deviations from the linear behaviour (effect of the protein geometry). Moreover, and as expected, the radial and tangential resolution radial averages curves are essentially the same curve.

Taking into account this information, a linear model is proposed for the loss of resolution, $R_\sigma(r)$ in terms of the radius for the radial average curves as it follows

$$R_\sigma(r) = R_0 + K(\sigma)r,$$

where r represents the radius, σ the angular uncertainty or error, and R_0 and $K(\sigma)$ the intercept term and the slope (that depends on the angular uncertainty σ). Hence, a linear regression to the radial and tangential radial averages was carried out for each reconstructed maps with $\sigma = 1, 1.5, 2, 2.5, 3$, the goal is to relate these uncertainties with the slope of the radial average. In Tables. 1 and 2 the slopes and intercept terms of the linear fittings for the $\beta-$galactosidase and the ribosome are respectively summarized. Considering the effect of the protein geometry, the regression were carried out in the intervals $[20, 0]$ px and $[20, 60]$ $\beta-$galactosidase and the ribosome respectively. It is observed that the higher slope, the higher angular error. In contrast, the intercept terms seems to be constant.

Finally, we wanted to determine the exact relation between the slopes and the committed error in the alignment process. Unfortunately, this seems to depend on the specific macromolecule. However, a simple linear model can also be proposed for which

$$K = m\sigma, \tag{1}$$

σ	K_r	$R_{0,r}$	R^2	K_t	$R_{0,t}$	R^2
1.0	0.0120	4.77	0.98	0.0094	4.84	0.90
1.5	0.0245	4.57	0.99	0.0186	4.72	0.97
2.0	0.0334	4.43	0.99	0.0321	4.41	0.99
2.5	0.0502	4.23	0.99	0.0489	4.17	0.99
3.0	0.0658	4.03	0.98	0.0562	4.18	0.99

Supplementary Table 1 – Summary of the linear fitting of the radial and tangential radial average curves of the $\beta-$galactosidase supplementary example. The table shows the slope K, the intercept term, R_0 and the coefficient of determination R^2. The subindex r and t denotes the radial and tangential components.

σ	K_r	$R_{0,r}$	R^2	K_t	$R_{0,t}$	R^2
1.0	0.0149	4.25	0.98	0.0118	4.39	0.99
1.5	0.0237	4.18	0.99	0.0193	4.34	0.99
2.0	0.0354	4.03	0.99	0.0296	4.22	0.99
2.5	0.0435	4.06	0.99	0.0377	4.24	0.99
3.0	0.0498	4.11	0.98	0.0437	4.27	0.99

Supplementary Table 2 – Summary of the linear fitting of the radial and tangential radial average curves of the Ribosome supplementary example. The table shows the slope K, the intercept term, R_0 and the coefficient of determination R^2. The subindexes r and t denotes the radial and tangential components.

with m a proportionality constant and K the measured slope from the radial average curves (radial or tangential). This linear fitting can be seen in Suppl. Fig. 8, and it obeys the linear equations $K = 0.0267\sigma - 0.0161$ and $K = 0.0179\sigma - 0.0024$ for the $\beta-$galactosidase and the ribosome respectively. Despite these linear regressions seems to be enough good, their slopes, m are different ($m = 0.0267$ and $m = 0.0179$), and therefore a hidden mechanism, probably some form of yet unknown normalization, must finally establish the exact relation between the slope of the radial averages curves and the committed error int he alignment process. The analysis of this hidden mechanism will be part of a future work.

1 Supplementary figures

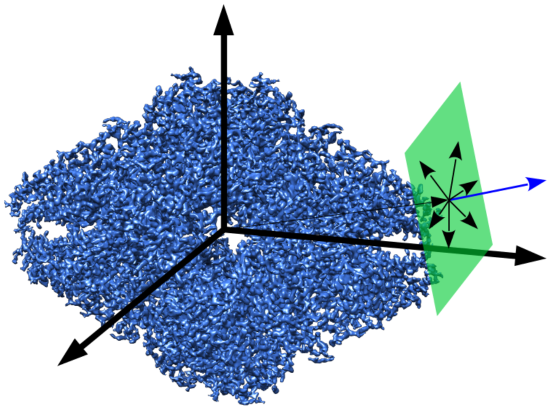

Supplementary Figure 1 – **(Supplementary) Radial an tangential resolution measurement**. For each voxel, the radial vector and a set of tangential vector are calculated. The local resolution corresponding to those vectors are calculated. By averaging the tangential resolutions the tangential component is calculated.

Supplementary References

[1] C. O. S. Sorzano, J. Vargas, J. Oton, V. Abrishami, J. M. de la Rosa-Trevin, J. Gomez-Blanco, J. L. Vilas, R. Marabini, J. M. Carazo, "A review of resolution measures and related aspects in 3D Electron Microscopy", Progress in Biophysics and Molecular Biology, **124**, 1-30 (2017).

[2] J. L. Vilas, J. Gomez-Blanco, P. Conesa, R. Melero, J. M. de la Rosa Trevın, J. Oton, J. Cuenca, R. Marabini, J. M. Carazo, J. Vargas, C. O. S. Sorzano, "MonoRes: automatic and accurate estimation of local resolution for electron microscopy maps", Structure 26, 337–344, (2018).

[3] Y.Z. Tan, P.R. Baldwin, J.H. Davis, J.R. Williamson, C.S. Potter, B. Carragher, D. Lyumkis, "Addressing preferred specimen orientation in single-particle cryo-EM through tilting", Nature Methods, 14, 793-796 (2017).

[4] A. Bartesaghi, A. Merk, D. Matthies, X. Wu, J. Milne, S. Subramaniam, "2.2 ÅResolution Cryo-Em Structure of Beta-Galactosidase in Complex with a Cell-Permeant Inhibitor", Science, 348, 1147 (2015).

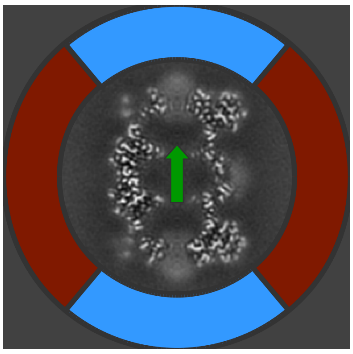

Supplementary Figure 2 – **(Supplementary) Scheme of MonoDir noise estimation area**. Given a measurement direction (green arrow), the noise in calculated in a shell greater than the particle radius. To avoid directional filtering artifacts only the intersection of a cone along the filtering direction with the shell is used to estimate the noise (blue area)

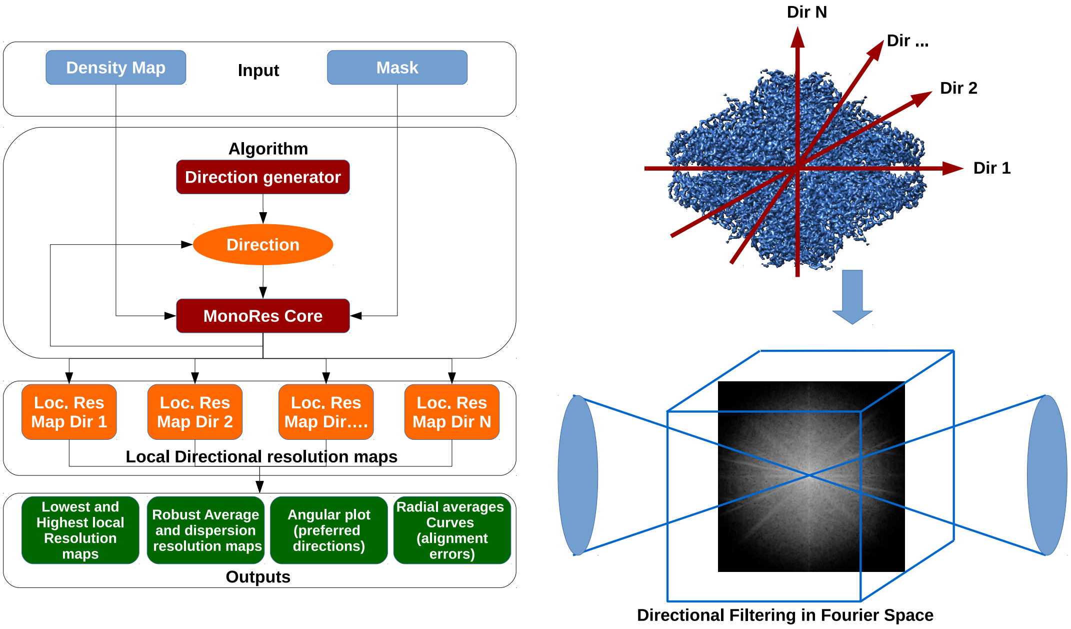

Supplementary Figure 3 – **(Supplementary) Scheme of MonoDir local-directional resolution estimation**.

[5] M. Fislage, J. Zhang, Z.P. Brown, C.S. Mandava, S. Sanyal, M. Ehrenberg, J. Frank, "Cryo-EM shows stages of initial codon selection on the ribosome by aa-tRNA in ternary complex with GTP and the GTPase-deficient $EF-Tu^{H}84A$", Nucleic Acids Research, 46, 11, 5861-5874, (2018)

[6] C.O.S. Sorzano, J. Vargas, J. Oton, V. Abrishami, J.M. de la Rosa-Trevin, S. del Riego, A.

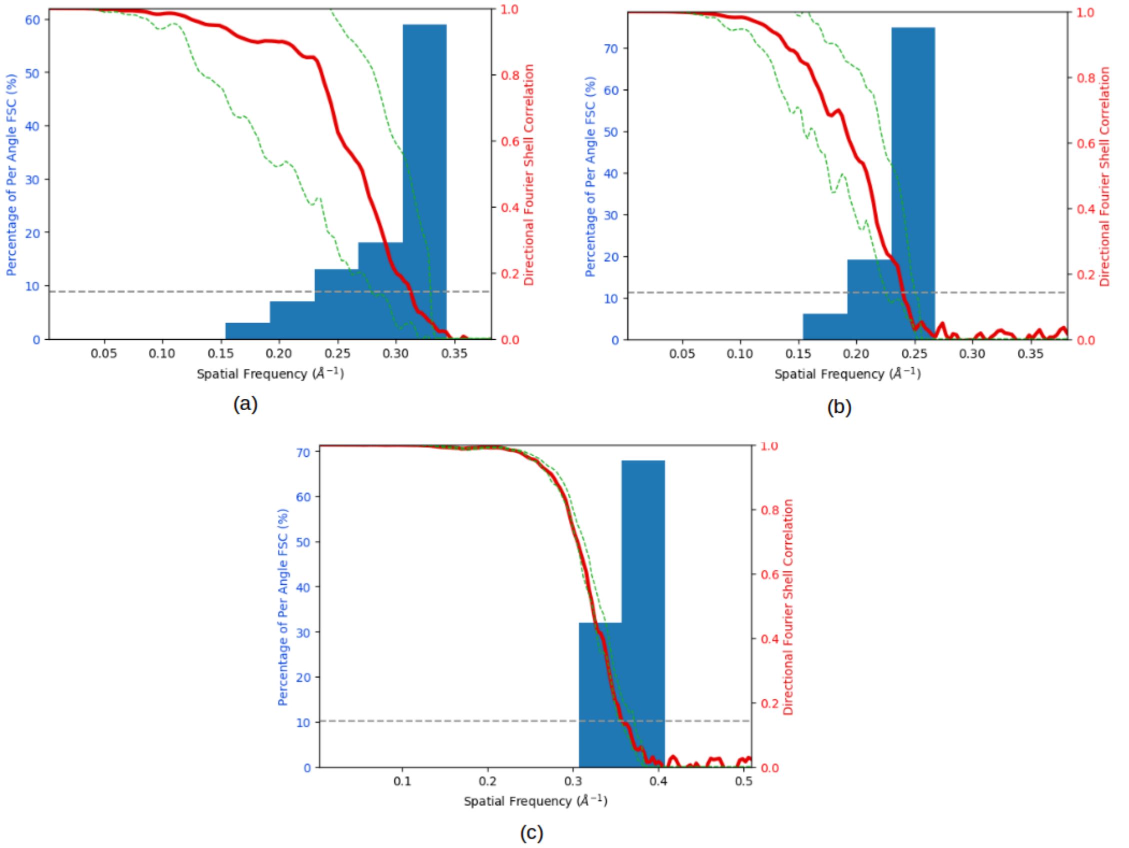

Supplementary Figure 4 – **(Supplementary) Results of the 3DFSC [3]** for the Influenza Hemagglutinin (HA) trimer when the sample is (a) untilted,(b) tilted 40 degrees, and (c) The proteasome 20S.

Fernandez-Alderete, C. Martinez-Rey, R. Marabini, J.M. Carazo, "Fast and accurate conversion of atomic models into electron density maps", AIMS Biophysics, 2, 20150102, (2015)

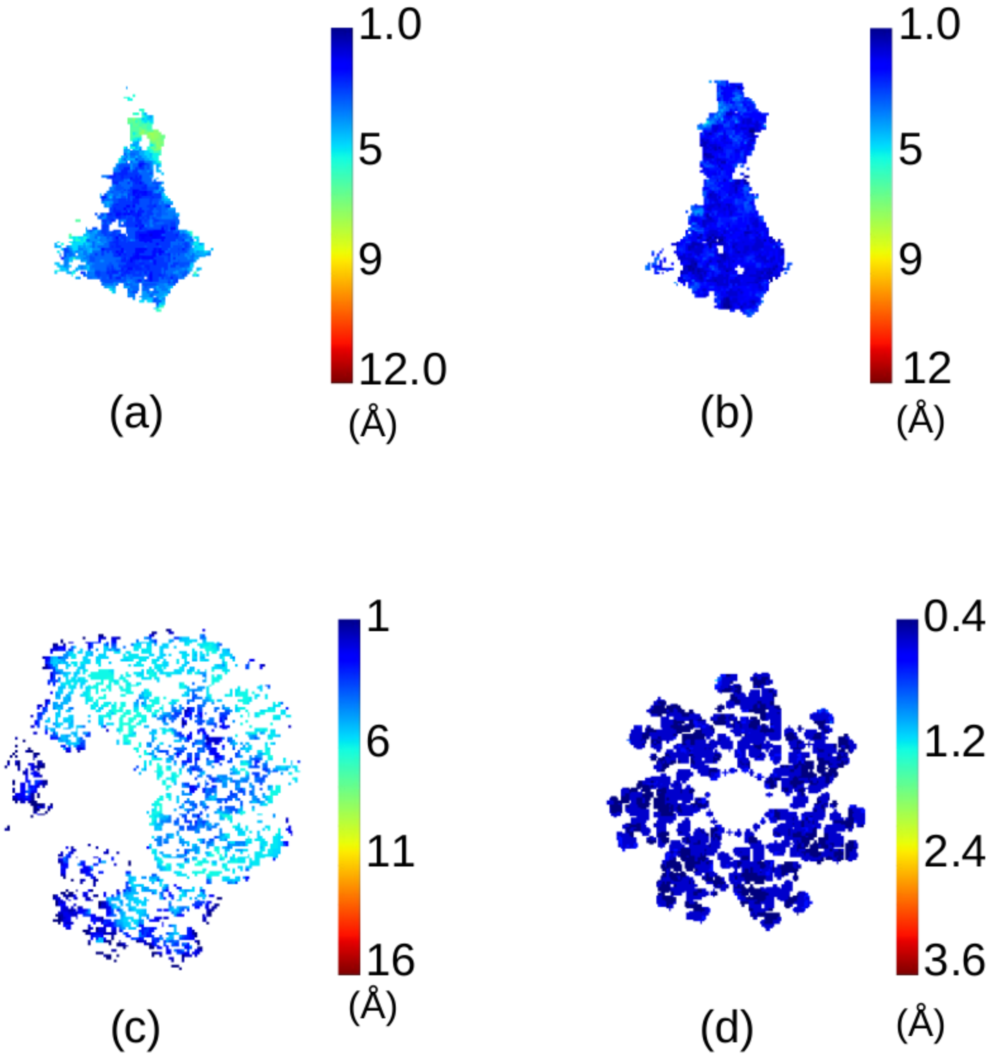

Supplementary Figure 5 – **(Supplementary) Interquartile measures of local-directional resolution for Influenza Hemagglutinin (HA) trimer** when the sample is (a) untilted,(b) tilted 40 degrees (c) ribosome 70S and (d) proteasome 20S.

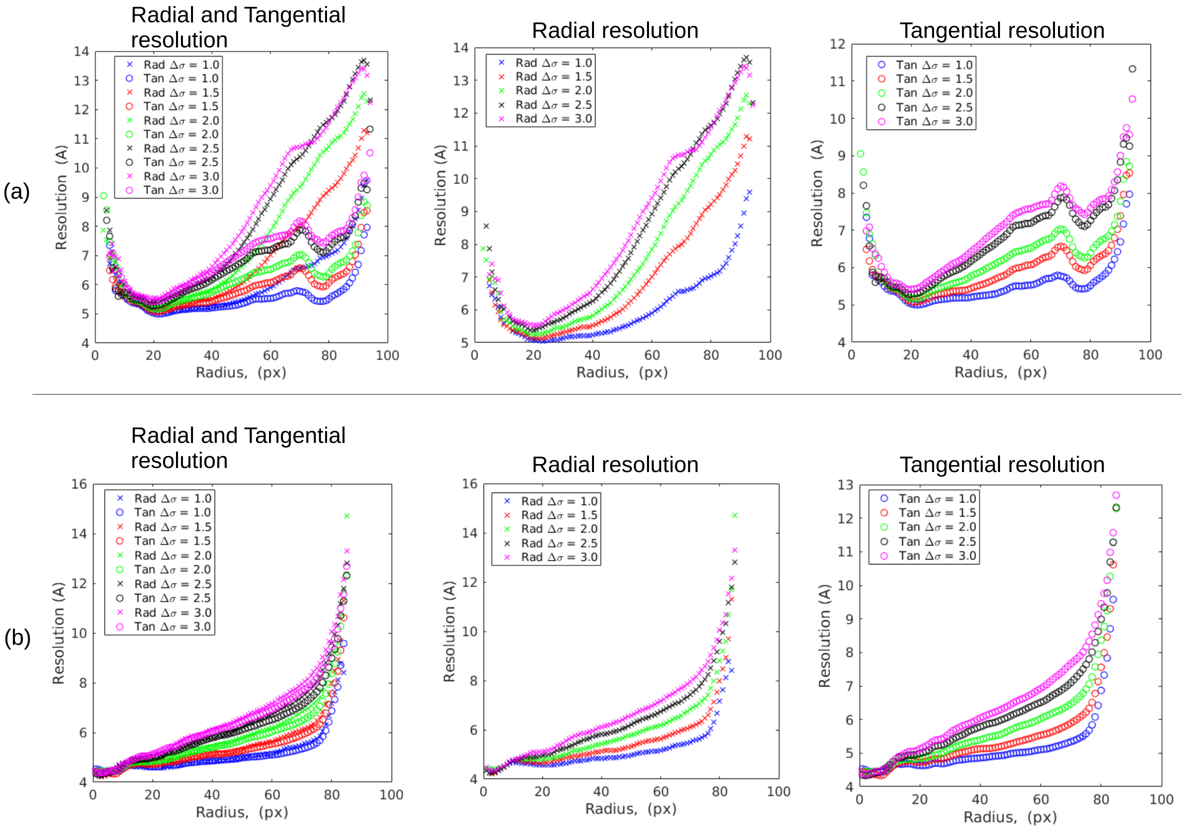

Supplementary Figure 6 – **(Supplementary) Radial average curves of the radial (Rad) and tangential (Tan) resolution considering several angular errors** with $\sigma = 1, 1.5, 2, 2.5, 3$ degrees, for the (a) and $\beta-$galactosidase map from pdb entry $5a1a$ and (b) Ribosome map from pdb entry $5wf0$.

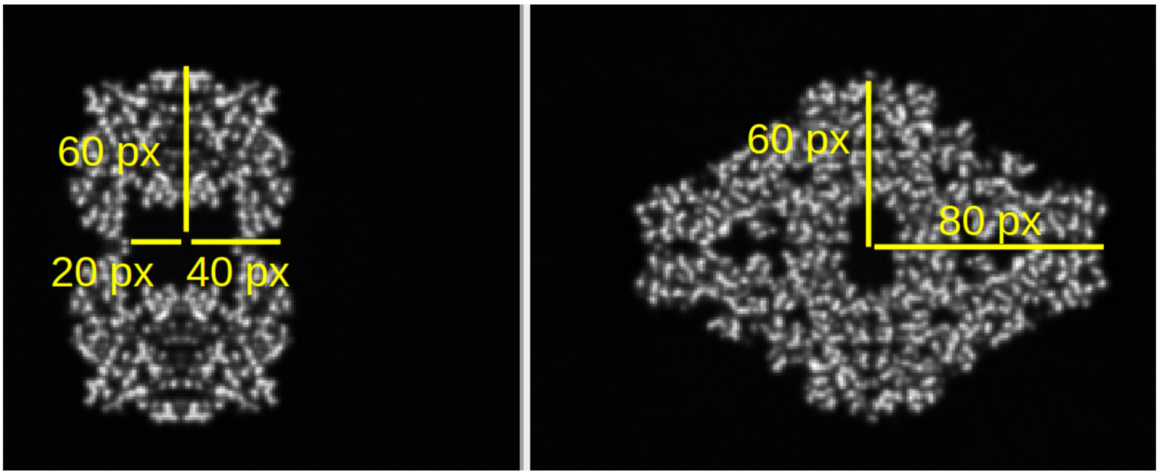

Supplementary Figure 7 – (Supplementary) **Central slices of the $\beta-$galactosidase map** from pdb entry $5a1a$ with the main distances in pixels that determine its geometry.

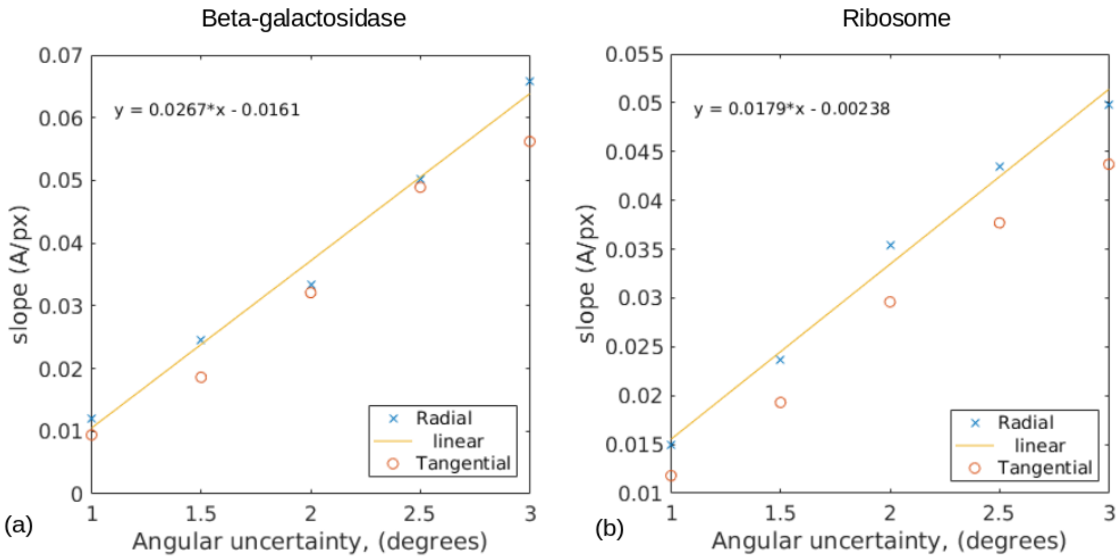

Supplementary Figure 8 – **(Supplementary) Linear fitting of the pairs** (K, σ) for the (a) $\beta-$galactosidase and (b) the Ribosome.
